# Extracting Teeth, and the Indications Therefor

**Published:** 1873-03

**Authors:** Louis Aguspath

**Affiliations:** Helana, Ark.


### Extracting Teeth, and the Indications Therefor.

BY LOUIS AGUSPATH, HELANA, ARK.

Having the honor to be appointed as one of the committee to report on "Indications for, and Extracting Teeth," and thinking it probable that no other member has taken hold of the subject, I have concluded to say a few words in regard to this very important point of dental practice. Still, at the same time, I feel that, perhaps, I am the least competent to handle the subject in the manner it justly deserves. It is indeed singular how little attention this subject has received from the great lights and abler writers on dental science. They seem to have given no general law to guide the student and less skillful practitioner.

Knowing however my own deficiency, I shall not attempt to explain *when* and *how* teeth ought to be extracted, but shall only give some of my thoughts on the subject, in order to open the way fairly for discussion, and a careful consider ation of the question.

As there is hardly a practitioner who is not called upon daily to perform this painful operation, I hope that every member of the Association will freely participate in the discussion.

Following the natural order of the topic, we shall first consider the indications for the extraction of temporary teeth. To come to a correct conclusion as to when we should, and when we should not extract temporary teeth, it is important that the operator should be thoroughly acquainted with the periods of the normal eruption of the different permanent teeth.

In the majority of cases where the child is of healthy and robust constitution, nature will perform all that is necessary. But in children who are weakly or sickly, the dentist's skill will be required either to assist nature in her efforts or to direct the eruption of the permanent tooth.

Tendency to irregularity of the permanent teeth is one of the first and principal indications for the removal of temporary teeth. Again, should the crown of the temporary tooth have been destroyed by caries to such an extent as to bring the rough edges of the root in contact with the soft tissue, and thus cause irritation, ulceration, and sloughing of the same, or set up inflammation of the periosteum, exfoliation of the alveolar process, and alveolar abscess, then, and then only, is the operator justifiable in removing teeth or roots previous to the period for the eruption of the permanent ones.

The indications for the removal of temporary teeth, with the exception perhaps of some isolated cases, as a general rule is very plain, and with sufficient knowledge of the physiology of the teeth, the operator will very rarely be led into errors.

It is not so with the permanent teeth; here the dentist is well aware that if a tooth is once lost, nature will not replace the lost member, and thus an error here committed can not be corrected. I shall now enumerate a few indications for extraction; but to decide upon the propriety of the removal of a permanent tooth, will greatly exercise the judgment, and depend upon the experience of the operator.

Among the leading indications for extraction are teeth causing diseases of the antrum, facial neuralgia, dead teeth and roots, teeth with incurable abscess, loose teeth, and in fact all the teeth that have become a source of irritation to the surrounding tissues, and where the diseased tooth gives no hope of restoration to health. On the above points I shall not elaborate, but leave them open for the discussion of those more able, and of more experience.

The extraction of teeth, although in the majority of cases simple and safe, is by no means to be considered altogether free from accidents. Few of us, perhaps, have been so fortunate as never to meet with some, however slight. Who of you gentlemen has not at some time, in extracting a tooth brought away a part of the alveolar process with it? or extracted two teeth, intending to remove but one? The latter accident happened to the writer in his early practice. While extracting the first lower molar the second bicuspid was brought away also. You may imagine my feelings when I inform you that the sufferer was a beautiful young lady of my acquaintance, and I at the same time unmarried. But these accidents may be almost entirely avoided, if the operator has sufficient knowledge of the parts to be operated upon, and if he is provided with suitable instruments, and the same are used with a skillful hand.

The extraction of teeth is perhaps the most painful operation for the time being that the surgeon can perform, and as Dr. Harris says, "there are few cases in surgery that cause more dread than the extraction of a tooth."

Considering those two points alone, the important question of "time" will at once present itself. We all admit the severity of the pain we inflict. Then in how long or short a time should the operation be performed?

Some good operators advise us to extract teeth slowly, as the patient will, they say, bear the pain better, and with less shock to the nervous system. However correct they may be in their theory, the writer's opinion is in direct opposition. We say if you are compelled to inflict pain, do it quickly, and get through in as short a time as possible. In proof of the correctness of my position, I would ask—Have either of you ever been shot? If so, did the penetration of the bullet cause you much pain? Or perhaps you may unexpectedly have cut yourself. Did you experience much suffering? Try and inflict these injuries slowly, and with a knowledge that it is to be done, and you will find that the result will be a decidedly different one.

To fortify my argument better, I shall furnish one other illustration. If you have ever received a blow upon the ulnar nerve, where it crosses the elbow joint, the place commonly called the "funny bone," you will remember that it destroyed almost every particle of sensibllity in the lower arm. Now with this fact before us, we assert that if it were possible to strike a blow upon the trunk of the maxillary nerve, we would be able to extract every tooth with comparatively no pain.

Basing my argument upon these points, I claim that my theory, if not altogether correct, is at least very plausible, and worthy of careful consideration. It has been the practice of the writer to operate as quickly as possible, and in many instances he has succeeded in removing three and even more teeth, the patient giving utterance to pain only when the first one was extracted; especially has this been true when the teeth to be removed were joining. This will generally prove the case in most instances, if the operation is rapidly performed.

With these remarks, gentlemen, I will leave this portion of the subject to your careful consideration, and will next proceed to give my mode of extracting a few teeth.

Imagine the root of a central incisor badly decayed, and which probably had previously a pivot tooth ingrafted upon it, the dentine lost, cementum brittle, with a firm alveolar attachment. Now in this case we make a perpendicular incision through the gum in the center of the root, and dissect the gums in two flaps, then take a sharp enamel chisel, and remove a part of the alveolar process, sufficient to enables us to take firm hold of the root with plain root forceps, with thin beaks, and with inward and outward movements, the latter the stronger, remove the root. In cases like the above, I object to the use of the duck-bill, forceps, as in most such cases it will be almost impossible to cut through the alveolar process, without at the same time either cutting through the root or fracturing the same, and thus making the operation more difficult, and giving the patient unnecessary pain. In the extraction of cuspids with strong attachments, I adopt the same method.

For badly decayed lower molar teeth, I prefer the cow-horn forceps, placing the same in the center of the tooth, and forcing the beaks by the closing pressure between the roots, and with a steady outward movement at the same time. Should the tooth at the bifurcation not be strong enough to resist the pressure and split, you will have the satisfaction in most instances of bringing out one root, and at the same time breakimg up the attachment of the other, which may then be taken out with root forceps, or a strong excavator if you please, without further trouble to the operator, or pain to the patient. Should the case in question present itself with roots spreading, or approximating at the apex, and thus inclose a thick, and firm septum, and with the crown too strong to be broken with the cow-horn forceps, I then make use of the splitting forceps, and remove each root singly.

One more case, gentlemen, and I will try your patience no longer. The extraction of the "*dentes sapientiœ*," especially the inferior ones, is frequently a source of great annoyance to the operator. This tooth we extract, with Physick's forceps, whenever circumstances will allow the use of the same; if not applicable, we use Harris's forceps for lower molars, in the usual manner. In connection with this subject I, would have been pleased to say a few words in regard to the wholesale extraction of teeth, to keep a number of rubber-boilers from starving, but want of time, and the disgust which I entertain for the same, forbid.

Thanking you gentlemen for your kind attention. I hope that you will discuss this subject in a manner commensurate with its importance.

